# Derivation and Validation of a Food Inflammatory Index Calibrated to Brazilian Dietary Patterns in Adults After Acute Myocardial Infarction

**DOI:** 10.7759/cureus.109280

**Published:** 2026-05-20

**Authors:** Andreia R Dias, Angela C Bersch-Ferreira, Rachel H Vieira Machado, Erlon O de Abreu-Silva, Aline Marcadenti

**Affiliations:** 1 Graduate Program in Public Health, Faculdade de Saúde Pública da Universidade de São Paulo (FSP-USP), São Paulo, BRA; 2 Department of Education and Research, Beneficência Portuguesa de São Paulo (BP), São Paulo, BRA; 3 Hcor Research Institute (IP-Hcor), Hospital do Coração, São Paulo, BRA; 4 Division of Health Care Sciences, Dresden International University, Dresden, DEU; 5 Graduate Program in Health Sciences (Cardiology), Instituto de Cardiologia/Fundação Universitária de Cardiologia do Rio Grande do Sul (IC/FUC), Porto Alegre, BRA

**Keywords:** cytokines, diet, inflammation, myocardial infarction, regression analysis

## Abstract

Background: The inflammatory potential of the diet modulates immune pathways implicated in residual cardiovascular risk after acute myocardial infarction (AMI). Because dietary patterns differ across populations, culturally calibrated tools are needed to accurately assess the inflammatory properties of habitual intake. We aimed to derive a food group-based Food Inflammatory Index (FII), anchored to a multimarker inflammatory panel, in Brazilian adults undergoing secondary prevention and to evaluate its construct validity.

Methods: This cross-sectional analysis included baseline data from 170 adults enrolled two to six months after AMI in a dietary intervention trial. Dietary intake was assessed using a validated food frequency questionnaire and aggregated into 31 food groups. Seven plasma biomarkers (interleukin (IL)-2, IL-4, IL-6, IL-10, tumor necrosis factor alpha (TNF-α), interferon gamma (IFN-γ), and C-reactive protein (CRP)) were standardized and combined into a composite inflammatory gradient. Reduced-rank regression identified dietary patterns that maximized the explained variance in this gradient. Backward stepwise linear regression was then applied to derive food group weights for the FII, with higher scores indicating a more pro-inflammatory dietary profile. Biological coherence was examined using correlation and multivariable regression analyses.

Results: Participants were predominantly male (74.1%, n = 126), with a mean age of 58.6 ± 9 years and a mean body mass index (BMI) of 28.5 ± 4.4 kg/m². The median FII was 1.82 (interquartile range (IQR): 0.74-3.16). Weak but statistically significant correlations were observed between the FII and TNF-α (ρ = 0.16, p = 0.035) and IFN-γ (ρ = 0.15, p = 0.049); however, these associations were no longer significant after adjustment for age, sex, BMI, physical activity, and energy intake.

Conclusions: An empirical FII calibrated to Brazilian dietary patterns in adults after AMI was derived using a multibiomarker inflammatory approach. The index showed modest evidence of biological coherence through weak unadjusted associations with selected inflammatory biomarkers, but these associations were not sustained after multivariable adjustment. Therefore, findings should be interpreted as exploratory and hypothesis-generating. Further longitudinal and external validation studies are necessary to determine the reproducibility, predictive validity, and clinical applicability of the proposed index.

## Introduction

Low-grade inflammation is a chronic state of immune activation associated with adverse cardiometabolic outcomes, including atherosclerotic cardiovascular disease (ASCVD) [1]. In secondary prevention, individuals in the post-acute myocardial infarction (AMI) phase often exhibit residual inflammatory risk despite optimal lipid control [2]. This is clinically relevant, as approximately 20% of patients experience a recurrent cardiovascular event within one year [3]. Dietary patterns represent modifiable risk factors for ASCVD and can modulate inflammatory biomarkers such as C-reactive protein (CRP), interleukin (IL)-6, and tumor necrosis factor-α (TNF-α) [4]. In this context, there is a clear need for clinically applicable tools to quantify the inflammatory potential of the diet in this high-risk population.

Two main approaches have been used to assess dietary inflammatory potential. The Dietary Inflammatory Index (DII®) is literature-derived and links nutrients and foods to inflammatory markers [5-7]. In contrast, empirical indices such as the Empirical Dietary Inflammatory Pattern/Index (EDIP/EDII) are derived from observational data using circulating biomarkers to identify food group combinations predictive of inflammation [8]. Although methodologically robust (the DII is a score derived from a systematic review, and the EDIP/EDII is based on the Reduced Rank Regression (RRR) methodology), these tools present limitations for routine clinical use and cross-cultural applicability. The DII relies heavily on isolated nutrients and external evidence, potentially overlooking food matrix effects, whereas the EDIP/EDII was developed in North American cohorts, which may limit its generalizability to other populations [9-11]. Moreover, the association of these indices with systemic inflammation in the context of secondary cardiovascular prevention remains uncertain [12,13].

The Brazilian dietary context underscores the need for locally calibrated instruments. Traditional staples such as rice and beans coexist with increasing consumption of ultra-processed foods, creating a distinct dietary profile [14]. Although empirical inflammatory indices have been developed in Brazil [10,15], these were validated mostly in primary cardiovascular prevention settings and were based on a limited biomarker panel. A gap remains for tools specifically calibrated to the metabolic complexity of secondary prevention, particularly among individuals with established ASCVD, a population with suboptimal adherence to cardioprotective dietary targets [16,17].

To address this gap, our study aimed to derive and preliminarily validate an empirical Food Inflammatory Index (FII), anchored in a seven-biomarker panel [18], and calibrated for Brazilian adults in the post-AMI setting. We hypothesize that higher FII scores may be associated with a composite inflammatory score, supporting its biological coherence.

## Materials and methods

Study design and population

A cross-sectional analysis was conducted using baseline data from the DICA-NUTS trial (Effect of the Brazilian Cardioprotective Diet and Nuts on Cardiometabolic Parameters in Post-acute Myocardial Infarction: a Randomized Clinical Trial), whose protocol [19], eligibility criteria [19,20], and primary results [20] have been previously published. Briefly, DICA-NUTS was a randomized, multicenter, superiority clinical trial that enrolled adults aged ≥40 years who had experienced a ST-segment elevation myocardial infarction (STEMI) or non-STEMI (NSTEMI) two to six months prior to enrollment. The primary objective of the DICA-NUTS study was to evaluate the effect of a Brazilian cardioprotective dietary pattern, with or without mixed nuts supplementation, on low-density lipoprotein cholesterol (LDL-c) levels in this population after 16 weeks of follow-up. For the present secondary analysis, 170 participants with complete data on dietary variables and all required inflammatory biomarkers were included [13].

The trial was registered at ClinicalTrials.gov (NCT03728127) and assigned the World Health Organization Universal Trial Number (WHO-UTN: U1111-1259-8105). The study was conducted in accordance with the ethical standards governing research involving human participants. Ethical approval was obtained from the Research Ethics Committee of Hospital do Coração (Hcor; coordinator center; approval number: CAAE 95382518.6.0000.0060 and number 2.826.317) as well as from the institutional review boards of all participating centers. Prior to enrollment, all participants provided written informed consent, which was obtained by trained study personnel.

Data collection

Demographic data, including age, sex, and self-reported race/ethnicity; clinical history (type of AMI, therapeutic procedures, comorbidities, and medication use); lifestyle features (smoking and alcohol consumption); and anthropometric measurements (weight, height, body mass index (BMI), waist, and hip circumferences) were collected following the study protocol [19,20]. Physical activity was assessed using the long form of the International Physical Activity Questionnaire (IPAQ), which has been translated and validated for Brazilian Portuguese [21].

Socioeconomic status was evaluated according to the Brazilian Criteria for Economic Classification [22] and subsequently converted to monthly household income to facilitate international comparability. Mean household income values for each category were converted from Brazilian reais (R$) to U.S. dollars (US$) using the June 2024 exchange rate (US$1 = R$5.45).

Dietary assessment and derivation of food groups

Dietary intake was assessed using a quantitative food frequency questionnaire (FFQ) previously validated for the Brazilian population [23]. To reduce measurement error and improve the accuracy of portion size estimation, a standardized photographic atlas of food portions was used during interviews.

Reported consumption frequencies and portion sizes were converted into grams per day (g/day) by standardizing frequency categories to a daily equivalent and multiplying by the corresponding portion weights. Portion weights were referenced to the FFQ’s standard serving size using predefined multipliers (small = 0.5×; medium = 1.0×; large = 1.5×; extra-large = 2.0×) [23]. Nutrient intake and total energy intake (TEI, in kcal/day) were estimated using specialized dietary analysis software (Sistema Vivanda de Alimentação®, São Paulo, Brazil) [24].

Subsequently, individual FFQ items were aggregated into 31 food groups based on nutritional similarity, following and adapting the grouping framework previously applied in the derivation of the inflammatory index in the Brazilian Estudo Longitudinal de Saúde do Adulto (ELSA-Brasil) cohort [15]. Nutritional similarity was defined by grouping food items with comparable macronutrient and micronutrient profiles and analogous roles within customary Brazilian dietary patterns [15]. To ensure cultural representativeness, the grouping scheme explicitly incorporated central components of the Brazilian diet, particularly the rice-and-beans matrix. For each participant, daily intakes (g/day) of individual items were summed within their respective food groups to compute total group consumption.

Culinary preparations and processed foods were retained in the classification to capture contemporary dietary behaviors and the current food environment. All dietary data were entered and processed according to a standardized protocol, with double verification for internal consistency. The plausibility of TEI was evaluated according to the criteria proposed by Willett [25]. Participants reporting implausible energy intakes (<500 kcal/day or >4000 kcal/day) were excluded from subsequent analyses [19]. TEI was included as a covariate in biomarker models and in analyses examining associations with the FII.

Construction of the FII

Seven plasma biomarkers representing both pro- and anti-inflammatory pathways were quantified: IL-2, IL-4, IL-6, IL-10, TNF-α, interferon gamma (IFN-γ), and CRP. Venous blood samples were collected into ethylenediaminetetraacetic acid (EDTA)-containing tubes, centrifuged (3,000 rpm for 15 minutes at 4 °C), and plasma aliquots were stored at -80 °C until analysis. Cytokines were quantified using a multiplex immunoassay panel (HCYTOMAG-60K-06, Merck®, Darmstadt, Germany), and CRP was measured by enzyme-linked immunosorbent assay (ELISA; E-EL-H5134, Elabscience®, Houston, TX, USA), according to the manufacturers’ protocols [13].

Each biomarker concentration was standardized as a z-score (mean = 0; standard deviation = 1). A composite biological score (hereafter referred to as the inflammatory gradient) was calculated as the arithmetic mean of the seven standardized biomarkers (each computed as the individual value minus the sample mean, divided by the sample standard deviation), thereby harmonizing differences in measurement units, numerical ranges, and variability across biomarkers. Equal weighting was intentionally adopted to avoid disproportionate influence of biomarkers with larger variances and to generate a biologically oriented composite response variable suitable for RRR, rather than to construct a latent psychometric scale.

To ensure that the composite score consistently reflected a pro-inflammatory state, z-scores for the anti-inflammatory/regulatory cytokines (IL-4 and IL-10) were multiplied by −1 to reverse their directionality. Consequently, higher values of the final composite score indicate a greater systemic inflammatory burden.

Derivation of the FII

RRR was applied using the 31 food groups (g/day) as predictor variables and the composite inflammatory gradient as the response variable. The first RRR factor, representing the linear combination of food groups that maximized the explained variation in the inflammatory gradient, was retained as the derived inflammatory dietary pattern.

The objective of this analytical strategy was not to estimate independent causal effects of isolated food groups, but rather to derive a multidimensional dietary pattern associated with a predefined inflammatory biomarker profile. In this context, food groups were interpreted as correlated components of real-world dietary behavior rather than independent etiological exposures.

Individual RRR factor scores were obtained by multiplying food group intakes by their corresponding RRR loadings. These factor scores were then used as the dependent variable in a multivariable linear regression model including all 31 food groups as independent variables. A backward stepwise selection procedure was applied to obtain a parsimonious and interpretable set of regression coefficients for the construction of the FII.

The regression-based recalibration step was intended to translate the RRR-derived dietary pattern into a weighted food group-based index, rather than to infer independent causal effects of individual foods. Therefore, the final coefficients were used as empirical weights representing the contribution of each food group to the inflammatory dietary pattern identified in this population.

For each participant, the FII was calculated as the weighted sum of the adjusted intake of the retained food groups (intake × β). To facilitate intuitive interpretation, the directionality of the score was standardized such that higher positive values consistently represented a more pro-inflammatory dietary profile.

This two-stage procedure, combining RRR derivation and regression-based recalibration, followed the conceptual framework of biologically driven dietary pattern analysis while adapting the score to the inflammatory profile and dietary structure of this secondary prevention population.

Construct consistency

The internal consistency of the index was evaluated across three complementary domains: (i) properties of the composite biological score, (ii) stability of the diet-inflammation mapping derived from RRR, and (iii) biological coherence of the associations between the FII and individual biomarkers.

Because each biomarker (IL-2, IL-4, IL-6, IL-10, TNF-α, IFN-γ, and CRP) was standardized as a z-score, their distributions were examined descriptively and formally assessed (including the Shapiro-Wilk test) to ensure adequate variability and to confirm that no single component disproportionately influenced the composite score.

Subsequently, two alternative RRR implementations were applied (Model 1: rrpack::rrr.fit; Model 2: rrr::rrr, type = “pca”) to evaluate the proportion of variance in the inflammatory gradient explained by RRR factor 1 and to assess the stability of the derived coefficients in terms of both magnitude and direction. When necessary, the overall sign of the factor was reoriented to preserve pro- versus anti-inflammatory interpretability. The objective of this step was not to compare dietary patterns, samples, or biomarker panels but rather to examine whether the FII remained stable across different statistical parameterizations of the same methodological framework.

Biological coherence was then evaluated using Spearman correlation coefficients and multivariable linear regression models, with the FII as the exposure and individual biomarkers (log-transformed) as outcomes. Models were adjusted for age, sex, BMI, physical activity, and TEI. Model assumptions and residual diagnostics were systematically assessed. All results were presented as point estimates with 95% confidence intervals (CI).

Reproducibility analyses

To assess reproducibility, the full derivation process of the FII was independently replicated using both RRR implementations (Model 1: rrpack::rrr.fit; Model 2: rrr::rrr, type = “pca”), maintaining the identical backward stepwise selection procedure. We compared the resulting regression coefficients (weights), proportion of explained variance, and factor orientation. The associations between the FII and inflammatory biomarkers were then re-estimated using identical modeling strategies, diagnostic criteria, and assumption checks.

Statistical analysis

Descriptive statistics, including means, medians, standard deviations, and quartiles, were calculated, and data distributions were visually inspected. A two-tailed α = 0.05 was adopted. Pearson correlation coefficients were applied to approximately normally distributed variables, whereas Spearman correlation coefficients were used for skewed or ordinal variables. Model assumptions were evaluated through residual analysis and influence diagnostics. RRR was implemented using dedicated R packages (rrpack/rrr) in R version 4.1.0, and the backward stepwise selection procedure was conducted using standard linear regression functions based on p-values. All analyses were conducted using R version 4.1.0 (R Core Team, 2021, R Foundation for Statistical Computing, Vienna, Austria). Primary analyses were based on complete cases; multiple imputations were not required, given the low proportion of missing data in key covariates.

## Results

Sample characteristics

Table 1 presents the characteristics of the evaluated sample, including the inflammatory profile. Three participants were excluded due to implausible energy intake, and five were excluded due to incomplete data availability. Among the 170 adults undergoing secondary prevention after AMI included in this analysis, 74.1% (n=126) were male. The mean age was 58.6 ± 9.0 years, the mean BMI was 28.5 ± 4.4 kg/m², and the median waist-to-hip ratio (WHR) was 0.97 ± 0.1. Additionally, 8.8% (n=15) of participants were current smokers. Diabetes, hypertension, and dyslipidemia were present in 25.9% (n=44), 60% (n=102), and 38.8% (n=66) of participants, respectively. The median inflammatory gradient was −0.12 (IQR −0.30 to 0.16).

**Table 1:** Sociodemographic and clinical characteristics of the sample (n=170). SD: standard deviation; MD: median; IQR: interquartile range; STEMI: ST-segment elevation myocardial infarction; NSTEMI: non–ST-segment elevation myocardial infarction *Brazilian reais were converted to United States Dollar (US$) at US$ 1 = R$ 5.45 (June 2024). ** In the post-myocardial infarction period, antihypertensive medications are used not only to control blood pressure but also in patients without a diagnosis of hypertension, as they act as cardioprotective therapy to preserve ventricular function, reduce myocardial stress, and improve prognosis.

Variable	n (%), mean (SD), or MD (IQR)
Age (years)	58.6 (9.0)
Male	126 (74.1)
Ethnicity	
White	116 (68.2)
Pardo	31 (18.2)
Other	23 (13.6)
Monthly household income (US$)*	
≥ $1,009	84 (49)
< $1,009	86 (51)
Smoking	
Former smoker	91 (53.5)
Current smoker	15 (8.8)
Alcohol consumption (g/day)	0 (0 – 1.5)
Physical activity	
Low levels	59 (34.7)
Moderate or high levels	111 (65.3)
ST-segment elevation myocardial infarction	105 (61.8)
Type of treatment	
Percutaneous coronary intervention	14 (8.2)
Percutaneous coronary intervention with a stent	133 (78.2)
Coronary artery bypass graft surgery	4 (2.4)
Optimal medical treatment	19 (11.2)
Previous comorbidities	
Hypertension	102 (60.0)
Dyslipidemia	66 (38.8)
Diabetes	44 (25.9)
Drugs in use	
Lipid-lowering medications	
Simvastatin	94 (55.3)
Atorvastatin	32 (18.8)
Lovastatin	1 (0.6)
Rosuvastatin	37 (21.8)
Ezetimibe	5 (2.9)
Fibrate	3 (1.8)
Antihypertensive medications**	170 (100)
Antiplatelet agents/Anticoagulant medications	170 (100)
Hypoglycemic medications	54 (31.8)
Body mass index (kg/m^2^)	28.53 (4.4)
Waist-hip ratio	0.97 (0.1)
Inflammatory biomarkers	
Interleukin-2 (pg/mL)	0.7 (0.6 – 0.8)
Interleukin-4 (pg/mL)	17.2 (3.8 – 50.6)
Interleukin-6 (pg/mL)	1.1 (0.9 – 1.7)
Interleukin-10 (pg/mL)	3.7 (2.3 – 6.5)
Tumor necrosis factor alpha (pg/mL)	8.5 (6.1 - 12)
C-reactive protein (pg/mL)	86.7 (54.3 – 130.7)
Interferon gamma (pg/mL)	1.9 (1.3 – 2.5)

Table 2 summarizes the distribution of consumers and usual food intake across all 31 food groups, as well as the FFQ items reclassified into these groups. Overall, the data indicate a mixed dietary pattern that combines elements of the traditional Brazilian dietary pattern with a substantial intake of processed foods.

**Table 2:** Reclassified food frequency questionnaire food items, percentage of consumers and daily intake across the 31 food groups. N: number of observations; %: percentage; M: mean; SD: standard deviation.

Food Group	Foods	Consumers		Intake (g/day)	
		N	%	M	SD
French fries	French fries or fried cassava; fried snacks	110	64.7	9.9	21
Tubers	Potato, cassava, yam (boiled or baked), mashed potatoes	160	94.1	36.2	36.6
Sandwich	Sandwiches (hot dog, hamburger)	71	41.8	6.6	14.1
Coffee with sugar	Coffee or tea	156	91.8	142.4	138.6
Chicken meat	Chicken (boiled, fried, grilled, roasted)	164	96.5	33.8	33.3
Fish meat	Fish (boiled, fried, roasted) and seafood	141	82.9	19.5	49.5
Pork meat	Pork (loin, chop)	103	60.6	10.8	16.2
Processed meat	Bacon and processed meats (ham, mortadella, sausage, frankfurter); traditional bean and meat dishes (e.g., feijoada, feijão tropeiro); industrialized hamburgers, nuggets, meatballs	133	78.2	19.5	36.5
Red meat	Beef (steak, boiled, roasted), offal, organ meats; dried or salted meat; dishes with meat in farofa; pasta with meat sauce, lasagna, gnocchi; soups with meat	167	98.2	85.4	66.8
Cereals	Cooked white or brown rice with oil and seasonings; plain cookies or industrial toast (sweet or savory); homemade cakes (plain or filled); cassava flour, couscous, oats, tapioca; farofa without meat; pasta with sauce without meat; French bread, sliced bread (white, whole grain, sweet), homemade toast; cooked or fried polenta; baked snacks	170	100	237.7	132
Beer	Beer	51	30	43	136
Chocolate	Chocolate powder (added to milk); dark chocolate; milk chocolate, bonbons	116	68.2	5	11.9
Industrial condiments	Industrialized condiments (mustard, ketchup, soy sauce)	61	35.9	0.3	0.6
Natural condiments	Natural condiments (parsley, cilantro, oregano, basil)	125	73.5	0.2	0.3
Milk-based sweets	Filled, wafer, or butter cookies; dulce de leche; homemade desserts (pudding, mousse, layered dessert); industrialized desserts (cakes, sweets)	133	78.2	12.4	22.1
Non-milk sweets	Homemade fruit preserves and sweets; honey, jam	65	38.2	2.9	7.6
Fruits	Avocado; banana; guava; orange, tangerine, pineapple; apple, pear; papaya; melon, watermelon	167	98.2	274.2	303.5
Legumes	Beans (pinto, red, black, green); chickpeas, soy; lentils, peas (without meat)	165	97.1	74.5	86.4
Milk and cheese	Skimmed milk or yogurt; whole milk or yogurt; semi-skimmed milk or yogurt; cheeses	166	97.7	147.3	145.4
Mayonnaise	Mayonnaise, ready-made salad dressing, pâté, whipped cream; mayonnaise-based vegetable salads	98	57.7	5.4	8
Margarine	Butter with or without salt, salted margarine, unsalted margarine	144	84.7	9	9.4
Other vegetables	Vegetables (carrot, chayote; excludes tubers); soups without meat	156	91.8	22.2	33.7
Eggs	Eggs (boiled, fried)	153	90	24.6	31.2
Pizza	Pizza, pancake	121	71.2	14.4	26.7
Vegetable oils	Oil, olive oil for salad dressing	114	67.1	2.6	3.1
Soft drinks	Soft drinks (regular, diet, light)	85	50	52.4	147.8
Artificial juice with sugar	Industrialized juice	59	34.7	64.1	149.5
Natural juice without sugar	Natural juice (including concentrate, pulp, and whole juice)	117	68.8	49.3	71.3
Tomato	Tomato	154	90.6	27.2	28.2
Cruciferous vegetables	Broccoli, cauliflower, leafy greens (kale, cabbage, Swiss chard)	154	90.6	21.4	19.4
Leafy vegetables	Lettuce	152	89.4	18	20.4

Construction of the FII

Appendix A presents the RRR model using the 31 food groups (g/day) as predictor variables and the inflammatory gradient as the response variable, with the RRR coefficients for each variable reported. In column 2, the coefficients were obtained using the rrr.fit function from the rrpack package (Model 1, representing the proposed FII). In column 3, the factor 1 coefficients were obtained using the rrr function from the rrr package, specifying type = "pca", which fits a principal component analysis (PCA) model as a special case of reduced-rank regression (Model 2, for the assessment of construct consistency and reproducibility). Appendix B presents a description of the dependent variable generated by multiplying the selected RRR factor 1 from each model (1 and 2) by the corresponding food intake.

Table 3 presents the coefficients (β) obtained from the linear regression model, considering the dependent variable the one generated by multiplying the selected RRR factor 1 by the corresponding food intake, and food consumption as the independent variable. All variables had a p-value below the criterion established in the stepwise backward procedure (<0.10); therefore, all remained in the final model. The median FII of the evaluated population, calculated as the weighted sum of all regression coefficients by the consumption of each food group (β × intake), was 1.82 (IQR 0.74-3.16).

**Table 3:** Regression coefficients and corresponding p-values from the linear regression (Model 1) including the 31 food groups as independent variables and the reduced rank regression factor 1/food consumption–derived variable as dependent variable.

Covariates (food groups)	Coefficients (β)	p-value
French fries	-0.00052	<0.001
Tubers	-0.00202	<0.001
Sandwich	0.00208	<0.001
Coffee with sugar	-0.00042	<0.001
Chicken meat	-0.00047	<0.001
Fish meat	-0.00109	<0.001
Pork meat	0.00256	<0.001
Processed meat	0.00049	<0.001
Red reat	0.00036	<0.001
Cereals	-0.00051	<0.001
Beer	-0.00006	<0.001
Chocolate	-0.00107	<0.001
Industrial condiments	0.04997	<0.001
Natural condiments	-0.13531	<0.001
Milk-based sweets	-0.00103	<0.001
Non-milk sweets	0.00808	<0.001
Fruits	0.00021	<0.001
Legumes	-0.0005	<0.001
Milk and cheese	0.00036	<0.001
Mayonnaise	-0.00105	<0.001
Margarine	0.00485	<0.001
Other vegetables	-0.00025	<0.001
Eggs	0.0021	<0.001
Pizza	-0.00216	<0.001
Vegetable oils	0.02089	<0.001
Soft drinks	0.00023	<0.001
Artificial juice with sugar	0.00024	<0.001
Natural juice without sugar	-0.00059	<0.001
Tomato	0.00155	<0.001
Cruciferous vegetables	-0.00221	<0.001
Leafy vegetables	0.00245	<0.001

Appendix C presents the regression coefficients obtained from Model 2, which used the PCA approach, indicating the construct consistency and reproducibility of the proposed FII. The median index calculated from Model 2 was 127.07 (IQR 77.52-163.52). Although the models differed substantially in scale, Model 1 (FII) exhibited lower variability (SD = 4.60) compared to Model 2 (SD = 109.65); these results support the construct validity of the proposed FII.

Biological coherence

No significant correlations were observed between the FII and IL-2, IL-4, IL-6, IL-10, or CRP. In contrast, Spearman’s rank correlation analyses demonstrated statistically significant positive associations between the FII and TNF-α (ρ = 0.162, p = 0.035), as well as between the FII and IFN-γ (ρ = 0.151, p = 0.049), although the strength of these associations was weak.

After adjustment for age, physical activity, BMI, sex, and TEI, the FII was no longer significantly associated with either TNF-α or IFN-γ (Table 4). For IFN-γ, BMI emerged as an independent predictor (β = 0.083; p = 0.028), indicating that greater adiposity was associated with higher IFN-γ concentrations, irrespective of dietary inflammatory potential.

**Table 4:** Association between the empirical Food Inflammatory Index and tumor necrosis factor alpha and interferon gamma, adjusted for potential confounding variables. TNF-α: tumor necrosis factor alpha; IFN-γ: interferon gamma. *R² = 0.082; AIC = 1011.19; **R² = 0.066; AIC = 734.76

	TNF-α (pg/mL)*			IFN-γ (pg/mL)**		
	β	95% CI	p-value	β	95% CI	p-value
Age (years)	0.085	0.005 - 0.165	0.038	0.008	-0.027 - 0.044	0.65
Sex	0.834	-0.823 - 2.502	0.32	0.170	-0.567 - 0.907	0.65
Physical activity	-1.765	-3.249 - -0.282	0.02	-0.569	-1.227 - 0.089	0.09
Body mass index (kg/m²)	0.158	-0.010 - 0.325	0.06	0.083	0.00904 - 0.15752	0.028
Energy intake (kcal/day)	0.00024	-0.00080 - 0.00128	0.65	-0.00031	-0.00077 - 0.00015	0.19
Food Inflammatory Index	-0.00032	-0.00336 - 0.00273	0.84	0.00063	-0.00072 - 0.00198	0.36

In contrast, for TNF-α, age (β = 0.085; p = 0.038) and physical activity (β = -1.765; p = 0.020) remained significant predictors.

## Discussion

In this secondary analysis of the DICA-NUTS study, we derived and preliminarily evaluated an empirical FII calibrated to Brazilian dietary patterns within the context of post-AMI. The methodological strategy employed is consistent with contemporary approaches in nutritional epidemiology that prioritize biologically plausible intermediary response variables [18]. RRR allows the identification of dietary patterns associated with pathways potentially linked to disease mechanisms and outcomes, thereby providing greater mechanistic coherence than purely exploratory dietary pattern methods [18,26]. Nevertheless, despite the conceptual rationale underlying the proposed index, the observed associations between the FII and inflammatory biomarkers were modest and attenuated after multivariable adjustment.

Brazilian dietary patterns are characterized by substantial heterogeneity, combining potentially protective foods with items commonly associated with Westernized and pro-inflammatory dietary profiles [10,14,15,27]. Such complexity may partially explain the coexistence of apparently opposing dietary signals within the derived index. Moreover, dietary exposures likely exert effects through multiple interconnected inflammatory pathways [28-30], which may modulate molecular mechanisms even in the absence of detectable changes in systemic inflammatory biomarkers [31]. In this regard, the use of a multimarker inflammatory panel incorporating both pro- and anti-inflammatory cytokines may represent a methodological strength compared with approaches relying on fewer biomarkers [8,15,18,26], although it still may not fully capture the complexity of residual inflammatory risk after AMI.

The consistency of food group loadings and coefficient directionality across distinct computational implementations of RRR suggests that the derivation procedure was methodologically reproducible within the present dataset. However, the proposed FII should be interpreted as an exploratory and internally derived construct rather than a formally validated dietary inflammatory index. The inflammatory gradient was intentionally constructed using equally weighted standardized biomarkers to harmonize differences in measurement scales and variances and to generate a biologically oriented response variable suitable for RRR, rather than to establish a latent psychometric construct. Consequently, formal reliability metrics such as Cronbach’s alpha or PCA-derived variance explanation were not originally performed. Likewise, although multicollinearity is intrinsically expected in dietary pattern analyses because food intake variables are highly intercorrelated, formal variance inflation factor analyses were not conducted. While these procedures are not routinely reported in previous RRR-derived dietary inflammatory index studies, their absence should be acknowledged as a methodological limitation.

Consistent with prior investigations evaluating dietary inflammatory indices [7,12,13,15], the correlations observed between the FII and inflammatory biomarkers were weak. After adjustment for BMI, physical activity level, and TEI, factors independently associated with systemic inflammation [32,33], the associations with TNF-α and IFN-γ were no longer statistically significant. This finding may reflect the multifactorial nature of residual inflammatory risk in post-AMI populations, in which inflammation is strongly influenced by adiposity, metabolic abnormalities, and pharmacological therapy [2,32,34-36]. In our sample, the high prevalence of diabetes mellitus, hypertension, dyslipidemia, and overweight may have contributed substantially to inflammatory variability. Furthermore, because participants were receiving real-world secondary prevention therapy, including statins and antiplatelet agents with recognized anti-inflammatory effects [35,36], biomarker variability may have been attenuated, thereby limiting the discriminative capacity of dietary inflammatory indices in this setting.

Several additional limitations should be considered. First, the cross-sectional design precludes causal inference and raises the possibility of reverse causality, particularly because participants may have modified their dietary habits following AMI diagnosis and nutritional counseling. Such post-event dietary modification may also partially explain biologically unexpected coefficient directions observed for some food groups. Second, dietary intake was assessed using an FFQ validated for the general Brazilian population, which may not have fully captured the nuances of dietary behavior in post-AMI individuals. Although standardized procedures, trained interviewers, predefined food-grouping criteria, and a photographic atlas were used to minimize information bias, FFQ-based dietary assessment inherently involves measurement error and subjectivity.

Third, the relatively modest sample size in relation to the number of food groups included in the derivation process raises concerns regarding overfitting, coefficient instability, optimism bias, and limited transportability of the derived index to other populations [37,38]. The inclusion of 31 food groups in a sample of 170 participants may also have increased susceptibility to type I error and limited statistical power after multivariable adjustment. Additionally, because the derivation and exploratory evaluation of the FII were conducted within the same dataset, external validation was not possible. Finally, pharmacological therapy was not fully standardized across participants; treatment adherence was not systematically assessed; and information regarding newer glucose-lowering agents with established cardiovascular and anti-inflammatory effects, such as sodium-glucose cotransporter-2 (SGLT2) inhibitors and glucagon-like peptide-1 (GLP-1) receptor agonists, was not specifically incorporated into the analytical models, representing potential residual confounding.

Our study also has several strengths. To our knowledge, the FII represents the first index specifically developed to assess the dietary inflammatory potential within the context of post-AMI. The data used to construct the index were obtained through rigorous and standardized collection procedures conducted by trained assessors using validated instruments. Although no independent associations between the FII and inflammatory biomarkers were observed in the present study after multivariable adjustment, its potential long-term clinical relevance cannot be excluded. This is particularly relevant considering prior studies conducted in Brazilian populations under both primary [15] and secondary cardiovascular prevention [39], which have demonstrated associations between dietary inflammatory indices and health outcomes. Collectively, these findings may support the biological plausibility of the proposed FII, despite the exploratory nature of the current results.

## Conclusions

We derived and preliminarily evaluated an empirical FII calibrated to Brazilian dietary patterns in adults after AMI using a multibiomarker inflammatory framework. Although the index demonstrated partial biological coherence through modest crude associations with TNF-α and IFN-γ, these associations were attenuated after multivariable adjustment, indicating limited independent biological association in this clinical context. Therefore, the findings should be interpreted as exploratory and hypothesis-generating rather than confirmatory. 

In addition, the cross-sectional design, relatively small sample size, potential multicollinearity among food groups, and absence of external validation limit broader inference and immediate clinical applicability. Future prospective studies with larger and independent cohorts, greater statistical power, formal validation procedures, and longitudinal assessment of biomarkers and clinical events will be essential to evaluate the reproducibility, predictive validity, generalizability, and potential clinical utility of the proposed index in the context of residual inflammatory cardiovascular risk.

## Appendices

Appendix A 

**Table 5:** Coefficients of the reduced rank regression models (RRR), considering food groups as predictor variables and the inflammatory gradient as the response variable. PCA: Principal Component Analysis.

	Model 1	Model 2
Covariates	RRR standard Factor 1	RRR “PCA” Factor 1
French Fries	-0.00052	0.00215
Tubers	-0.00202	-0.00184
Sandwich	0.00208	0.00360
Coffee with Sugar	-0.00042	-0.02976
Chicken Meat	-0.00047	0.01133
Fish Meat	-0.00109	0.05012
Pork Meat	0.00256	0.00145
Processed Meat	0.00049	0.00864
Red Meat	0.00036	0.06410
Cereals	-0.00051	0.18033
Beer	-0.00006	0.01610
Chocolate	-0.00107	0.00298
Industrial Condiments	0.04997	0.00006
Natural Condiments	-0.13531	0.00038
Milk-Based Sweets	-0.00103	0.00151
Non-Milk Sweets	0.00808	0.00111
Fruits	0.00021	0.93703
Legumes	-0.00050	0.19328
Milk and Cheese	0.00036	0.05424
Mayonnaise	-0.00105	0.00023
Margarine	0.00485	0.00035
Other Vegetables	-0.00025	0.00035
Eggs	0.00210	0.00545
Pizza	-0.00216	0.00258
Vegetable Oils	0.02089	0.00001
Soft Drinks	0.00023	0.06406
Artificial Juice with Sugar	0.00024	0.18294
Natural Juice without Sugar	-0.00059	0.05863
Tomato	0.00155	0.00945
Cruciferous Vegetables	-0.00221	-0.0031
Leafy Vegetables	0.00245	-0.00021

Appendix B 

**Table 6:** Descriptive characteristics of the dependent variable generated by multiplying the selected reduced rank regression factor 1 from each model (Model 1 and Model 2) by the corresponding food intake. Shapiro–Wilk test; M: mean; SD: standard deviation; MD: median; IQR: interquartile range. Different scales by construction; non-normal distributions (Shapiro–Wilk p < 0.001).

Dependent variable	M	SD	MD	IQR	p-value
Model 1	-0.007	0.19	-0.03	-0.10 – 0.09	<0.001
Model 2	350.07	317.29	308.91	138.91 – 431.64	<0.001

Appendix C 

**Table 7:** Regression coefficients and corresponding p-values from the linear regression (Model 2) including the 31 food groups as independent variables and the reduced rank regression factor 1/food consumption–derived variable as dependent variable.

Covariates (food groups)	Coefficients (β)	p-value
French Fries	0.00215	<0.001
Tubers	-0.0018	<0.001
Sandwich	0.0036	<0.001
Coffee with Sugar	-0.0298	<0.001
Chicken Meat	0.01133	<0.001
Fish Meat	0.05012	<0.001
Pork Meat	0.00145	<0.001
Processed Meat	0.00864	<0.001
Red Meat	0.0641	<0.001
Cereals	0.18033	<0.001
Beer	0.0161	<0.001
Chocolate	0.00298	<0.001
Industrial Condiments	0.00006	<0.001
Natural Condiments	0.00038	<0.001
Milk-Based Sweets	0.00151	<0.001
Non-Milk Sweets	0.00111	<0.001
Fruits	0.93703	<0.001
Legumes	0.19328	<0.001
Milk and Cheese	0.05424	<0.001
Mayonnaise	0.00023	<0.001
Margarine	0.00035	<0.001
Other Vegetables	0.00035	<0.001
Eggs	0.00545	<0.001
Pizza	0.00258	<0.001
Vegetable Oils	0.00001	<0.001
Soft Drinks	0.06406	<0.001
Artificial Juice with Sugar	0.18294	<0.001
Natural Juice without Sugar	0.05863	<0.001
Tomato	0.00945	<0.001
Cruciferous Vegetables	-0.0031	<0.001
Leafy Vegetables	-0.0002	<0.001
